# Beyond standard data collection – the promise and potential of BRAIN (Brain tumour Registry Australia INnovation and translation registry)

**DOI:** 10.1186/s12885-022-09700-3

**Published:** 2022-06-02

**Authors:** Lucy Gately, Katharine Drummond, Mark Rosenthal, Rosemary Harrup, Anthony Dowling, Andrew Gogos, Zarnie Lwin, Ian Collins, David Campbell, Elizabeth Ahern, Claire Phillips, Hui K. Gan, Iwan Bennett, Oliver M. Sieber, Peter Gibbs

**Affiliations:** 1 Personalised Oncology Division, The Walter and Eliza Hall Institute of Medial Research, 1G Royal Parade, Parkville, Melbourne, VIC 3052 Australia; 2 Department of Neurosurgery, Royal Melbourne Hospital, Parkville, VIC Australia; 3 Department of Surgery, University of Melbourne, Parkville, VIC Australia; 4 Medical Oncology, Royal Melbourne Hospital, Melbourne, VIC Australia; 5 Cancer & Blood Services, Royal Hobart Hospital, Hobart, TAS Australia; 6 Menzies Research Institute, University of Tasmania, Hobart, TAS Australia; 7 Department of Medical Oncology, St Vincent’s Hospital Melbourne, Melbourne, VIC Australia; 8 Department of Neurosurgery, St Vincent’s Hospital Melbourne, Melbourne, VIC Australia; 9 Department of Medical Oncology, Cancer Care Services, Royal Brisbane and Women’s Hospital, and The University of Queensland, Brisbane, QLD Australia; 10 Department of Medicine, Deakin University, Geelong, VIC Australia; 11 Department of Medical Oncology, University Hospital Geelong, Barwon Health, Geelong, VIC Australia; 12 Department of Oncology, Monash Health, Melbourne, VIC Australia; 13 School of Clinical Sciences, Monash University, Melbourne, VIC Australia; 14 Department of Radiation Oncology, Peter MacCallum Cancer Centre, Melbourne, VIC Australia; 15 Department of Medical Oncology, Austin Hospital, Heidelberg, VIC Australia; 16 Department of Medicine, University of Melbourne, Melbourne, VIC Australia; 17 Department of Neurosurgery, The Alfred, Melbourne, VIC Australia; 18 Department of Neurosciences, Monash University, Clayton, VIC Australia; 19 Department of Medical Biology, The University of Melbourne, Parkville, VIC Australia; 20 Department of Biochemistry and Molecular Biology, Monash University, Clayton, VIC Australia

**Keywords:** Brain tumour, Registry, Real-world data

## Abstract

**Background:**

Real-world data (RWD) is increasingly being embraced as an invaluable source of information to address clinical and policy-relevant questions that are unlikely to ever be answered by clinical trials. However, the largely unrealised potential of RWD is the value to be gained by supporting prospective studies and translational research. Here we describe the design and implementation of an Australian brain cancer registry, BRAIN, which is pursuing these opportunities.

**Methods:**

BRAIN was designed by a panel of clinicians in conjunction with BIOGRID to capture comprehensive clinical data on patients diagnosed with brain tumours from diagnosis through treatment to recurrence or death. Extensive internal and external testing was undertaken, followed by implementation at multiple sites across Victoria and Tasmania.

**Results:**

Between February 2021 and December 2021, a total of 350 new patients from 10 sites, including one private and two regional, were entered into BRAIN. Additionally, BRAIN supports the world’s first registry trial in neuro-oncology, EX-TEM, addressing the optimal duration of post-radiation temozolomide; and BioBRAIN, a dedicated brain tumour translational program providing a pipeline for biospecimen collection matched with linked clinical data.

**Conclusions:**

Here we report on the first data collection effort in brain tumours for Australia, which we believe to be unique worldwide given the number of sites and patients involved and the extent to which the registry resource is being leveraged to support clinical and translational research. Further directions such as passive data flow and data linkages, use of artificial intelligence and inclusion of patient-entered data are being explored.

**Supplementary Information:**

The online version contains supplementary material available at 10.1186/s12885-022-09700-3.

## Background

Real-world data (RWD) is increasingly embraced as a valuable source of information to address clinical and policy-relevant questions that are unlikely to be answered by clinical trials. RWD refers to the data collected through routine clinical care, that is outside conventional clinical trials [1]. The fundamental components include descriptors of patients, treatments and outcomes [2], and can offer unique insights into unmet clinical needs, pathways of care, and resource use. However, it may also include novel data sources, such as consumer data, that extend well beyond the medical record, adding richness and potential.

Traditionally, disease registries have collected a prospectively defined dataset, with information extracted at intervals from medical records, with the intent of supporting a broad range of research and audit efforts. Typically, these are stand-alone efforts with initial surface-level plans for output, rather than established with the intent of addressing specific research questions, as would be the case for a clinical trial. As such, it is not uncommon for important data points to be missing from the dataset, either because they were not collected, not well documented or not captured at all in the source data (typically the standard hospital record) including information that majorly impacts treatment and outcomes such as co-morbidities, treatment intent and performance status.

In Australia and internationally, RWD captured in registries has been increasingly utilised in observational analyses of epidemiology and burden of disease, treatment patterns, biomarkers and health outcomes of different treatments [3–11], as well as to support regulatory drug approvals [12, 13]. The most notable undertaking for brain cancer is the Austrian Brain Tumour Registry (ABTR), which has demonstrated the ability of RWD to contribute beyond retrospective reviews. Outputs have included the incidence of brain tumours and specific cancer subtypes in Austria [14, 15], as well as relative survival and outcomes of real-world, compared to clinical trial, populations [16]. More importantly, ABTR identified genetic findings to further subtype tumours with primitive neuroectodermal morphology [17], ultimately leading to a greater understanding of this tumour and enabled exploration of tailored therapeutic options [18]. Additionally, ABTR demonstrated the utility of FISH-based 1p19q testing in oligodendrogliomas [19], resulting in a standard set of procedures which have been incorporated into routine care.

Ultimately, the current excitement and enthusiasm regarding RWD’s potential is the added value that can be created such as using registries to support cancer drug development [20], translational research efforts (when combined with tissue-based analyses) and well-defined prospective studies. This prognostic and predictive information, derived from clinical, pathology and/or molecular data, is critical as we seek to move toward a personalised approach to management, potentially driving major gains in outcome through optimal use of available treatment options.

Currently, there is no clinical cancer registry dedicated to brain tumour patients in Australia. Given the relatively low incidence of brain tumours and the decentralisation of cancer treatment in Australia, it is difficult for any single Australian centre to accumulate sufficient numbers to generate the sample sizes required for impactful research. A multi-site prospective effort to collect comprehensive data has the potential to refine our understanding of brain tumours, including important differences between well-defined patient subsets, with varying prognoses and varying treatment responses, and could drive research to improve the quality and quantity of survival for patients with brain tumours.

Here we describe the development and implementation of a novel multi-site brain tumour registry enabling the collection of comprehensive data across a spectrum of clinical practices at a large number of Australian sites. This activity encompasses a variety of brain tumour types, including benign, primary and secondary tumours, as well as rare tumours that represent less than 1% of new brain cancer diagnoses each year.

## Methods

### Design

BRAIN (Brain tumour Registry Australia INnovation and translation) was designed to capture comprehensive clinical information about patients diagnosed with brain tumours from diagnosis through treatment and recurrence or death. Independent expert opinion was sought to determine the breadth and nuances of data collection, with multidisciplinary input from leads for neurosurgery (KD), neuro-oncology (MR, LG), and subspecialist radiation oncologists (CP). A single resource was determined crucial across benign, primary and secondary malignant brain tumours. To limit redundancy of data fields, the database was streamed into categories based on the WHO 2016 classification of brain tumours [21]. An 86-page data dictionary was defined, and the application was developed in collaboration with BIOGRID [22]. A summary of data fields is shown in Supplementary Table 1, noting that these can be amended or updated to incorporate changes in nomenclature or practices with time.

The creation of BRAIN leveraged an existing database at Royal Melbourne Hospital. This robust original single site registry was established in 2003 and had collected data on over 5000 patients. BRAIN expanded and refined the dataset and included multiple unique features, such as a patient registration model facilitating appropriate management of patient identifiers under a waiver of consent as well as making shared data visible across all treatment sites, allowing continuation of data capture across time and locations of care; completion and accuracy metrics; streamed data capture based on tumour type to avoid irrelevant fields; and an event-based system where data is collected at the time of major developments such as change in treatment strategy or progression. A modular design means clinical, biomarker, staging or other routine care data fields can be modified over time, including addition of new fields as these become relevant clinically or for evolving research technology (see Supplementary Fig. 1).

There is considerable experience within the authorship in multi-site national and international registries across eight other tumour types, including colorectal cancer [23] and pancreatic cancer. Similar to BRAIN, these have been established with the intent of supporting a broad range of collaborative research projects, linking datasets and research endeavours, rather than being traditional stand-alone data collection efforts.

### Development

BRAIN was developed in collaboration with BIOGRID [22]. BIOGRID provides a secure, 128-bit SSL encrypted, cloud-based platform that allows data to be entered directly, with clinicians or data officers accessing the electronic database via a webpage interface. This “smart” platform is designed to minimize data errors or missing data through features such as logic rules and built-in data definitions. Access is password protected and traceable, and individual sites can only view data on their own patients. A waiver of consent model was approved by the Human Ethics and Research Committee. De-identified data is accessible to researchers following application to BioGrid Australia, a process which includes ethical review by the internal Ethics Officer, scientific review by the BRAIN Management Committee, and individual data custodian approval from each site contributing to BRAIN.

### Implementation

Historical data from the original Royal Melbourne Hospital brain cancer registry was merged with BRAIN, with prospective collection occurring simultaneously for external testing and feedback. Further refinement of the interface, data fields and logic features were undertaken following this analysis, with subsequent development processes. BRAIN was then deployed for additional testing, before being steadily rolled out across multiple external sites. As an illustration for this article, an initial analysis of new patients entered between Feb 2021 – Dec 2021 was conducted for number of new patients entered, patient characteristics and breadth of tumour types, and data quality, with a focus on glioma as the most common primary brain cancer.

## Results

**Scheme 1:**
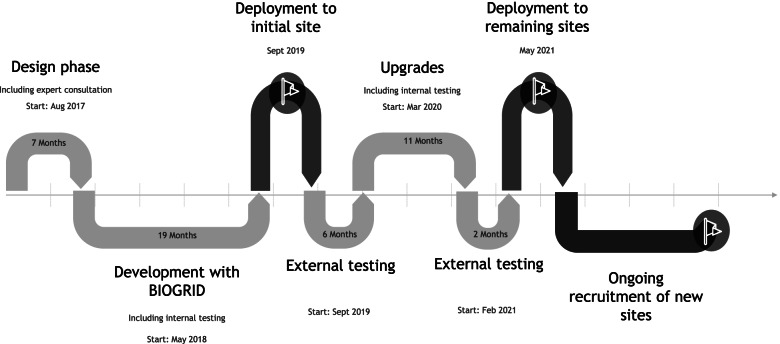
Timeline for BRAIN. Work commenced in August 2017 with the design phase. The development and testing phase (both internal and external) allowed for several revisions, taking 3 years before BRAIN (in its current state) was deployed to the remaining sites in May 2021

BRAIN was designed and developed between August 2017 and February 2021 (see Scheme 1). A breakdown of sites involved in BRAIN and its activities is shown in Table 1, demonstrating widespread involvement across metropolitan and regional Australia.

**Table 1:** Location of sites involved in BRAIN activities

**Location**	**Number of sites in BRAIN**	**Number of sites in EX-TEM**^**a**^	**Number of sites in BioBRAIN**^**b**^
Victoria			
Metropolitan	7	6	2
Regional	4	4	-
Interstate			
Tasmania	1	1	-
Queensland	-	3	-
New South Wales	-	2	-
Western Australia	-	1	-
International	1	-	-

^a^EX-TEM is a registry based randomised controlled trial examining the optimal duration of post-radiation temozolomide in newly diagnosed glioblastoma. This is described further below

^b^BioBRAIN is a translational project within BRAIN, initially focussed on patient derived tumour organoids (see below)

### Breadth of tumours and patient characteristics

Between February 2021 and December 2021, a total of 350 new adult patients were entered into BRAIN from 10 sites, including one private and two regional institutions, as they became activated. Logistics of patient identification and data entry varied by site with the majority utilising trained data officers or medical staff to examine multidisciplinary meetings, inpatient and theatre lists, as well as patient letters and private specialist rooms. Data was entered regularly at either weekly or monthly intervals with support for data collection provided in the form of an external data officer or per-patient payment. For sites with established internal data registries, migration was explored. For most sites, the focus was collecting consecutive patients with glioma, with tumour types beyond this collected depending on the individual site.

**Fig. 1:**
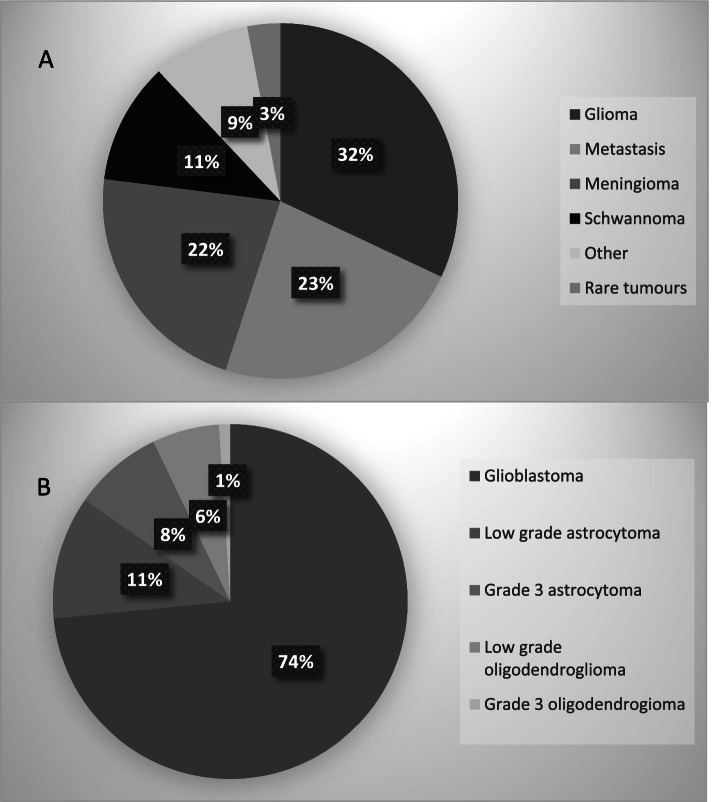
Breakdown of patients by **A** tumour type and **B** glioma subtype. Tumour types are presented in the legend in descending order. In part A, rare tumours include medulloblastoma and ependymoma with other representing additional tumour types not individually displayed such as spinal cord tumours, germ cell tumours and primary cerebral lymphoma. In part B, low grade tumours comprise grade 1 and grade 2 tumours

Figure 1 shows the breadth of tumour types collected with glioma (32%), metastases (23%) and meningioma (22%) being the most common. Within the metastatic subset, the most common primary pathology types were non-small cell lung cancer (23%), melanoma (14%) and breast cancer (14%).

Within glioma, the most common pathology was glioblastoma (72%), with 8% being grade 3 astrocytoma and 17% being low grade tumours. There was only one patient with grade 3 oligodendroglioma. Characteristics of these patients are shown in Table 2. Further analyses are underway to better understand these independent populations.

**Table 2:** Characteristics of patients with glioma

	**Low grade astrocytoma** ***n* = 13**	**Low grade oligodendroglioma ** ***n* = 7**	**Grade 3 astrocytoma** ***n* = 9**	**Glioblastoma** ***n* = 84**
Median age	33 years	40 years	54 years	64 years
**Gender**				
Male	9 (70%)	4 (57%)	4 (44%)	45 (54%)
Female	4 (30%)	3 (43%)	5 (56%)	39 (46%)
**Performance status**^**a**^				
ECOG 0–1	13 (100%)	7 (100%)	7 (78%)	62 (80%)
ECOG 2 +	0 (0%)	0 (0%)	2 (22%)	15 (20%)
**Extent of resection**^**b**^				
Biopsy	1 (8%)	1 (14%)	2 (22%)	18 (24%)
Subtotal resection	7 (54%)	3 (43%)	3 (33%)	29 (40%)
Gross resection	5 (38%)	3 (43%)	4 (44%)	26 (36%)
**Tumour biomarkers**				
IDH mutation	9 (73%)	7 (100%)	5 (55%)	7 (8%)
1p19q codeletion	ND	7 (100%)	ND	ND
**Adjuvant therapy received**				
Radiotherapy	2 (15%)	2 (29%)	6 (67%)	53 (63%)
Chemotherapy	2 (15%)	2 (29%)	4 (44%)	46 (55%)

*ND* Not done in majority of patients

^a^7 patients recorded as ‘unknown’

^b^9 patients recorded as ‘unknown’

### Tumour biomarker data

Relevant biomarkers are collected for all tumour types, including (but not limited to) hormone receptor/HER2 status for breast cancer metastases, WNT and SHH activation for medulloblastoma, and mitotic figures for meningioma. Standard glioma biomarker data collected into BRAIN includes 1p19q codeletion, IDH mutation, MGMT methylation, ATRX mutation and TERT promotor mutation with a free text field to capture other biomarkers of interest. A preliminary analysis demonstrated that 73% of low grade astrocytomas (*n* = 9), 55% of grade 3 astrocytomas (*n* = 5) and 8% of glioblastoma (*n* = 7) were IDH mutated, where data was available. The new WHO 2021 CNS tumour classification now places greater attention on IDH mutation status and focuses on within-tumour grading with the incorporation of novel molecular markers [24], meaning this data will be more complete over time. As such, those recorded as IDH mutant glioblastoma would now be reclassified as grade 4 astrocytoma with IDH mutation. Further updates are now underway to incorporate these. Additionally, all low grade oligodendrogliomas (*n* = 7) were IDH mutated and 1p19q co-deleted, consistent with the WHO 2016 classification [21] and the updated WHO 2021 classification [24].

### Data quality

Complete surgical and histopathology data, including the date and extent of surgery, the grade and histology subtype, was present for over 98% and ECOG performance status was recorded for 95% of the new cases (*n* = 350). Further quality audits are underway to assess capture of ongoing multidisciplinary therapy, particularly as such care is often provided across multiple sites. For example, a patient having initial surgery at a metropolitan site and then subsequent radiation and/or systemic therapy at a regional site, with follow-up often shared.

### Novel research activity – registry based randomised controlled trials (rRCTs)

Registry based trials are a new concept in cost efficient cancer research, offering the potential of recruiting large numbers of patients, using broad entry criteria that enable recruitment of a broad spectrum of real-world patients, and addressing important questions not being asked in conventional trials [25]. The initial rRCT leveraging BRAIN is EX-TEM, a study endorsed by the Australian Co-operative Trials Group for Neuro-Oncology (COGNO). This phase III study compares 6 vs 12 months of post-radiation temozolomide in patients with newly diagnosed glioblastoma. Patients are identified and consented at participating sites, with randomisation occurring centrally. All trial data is collected in BRAIN, including serious adverse events, with all patients continuing to be followed via the BRAIN registry after completing treatment and until death. EX-TEM is open at 16 sites, including a mix of private, public, metropolitan, and regional sites, and an international collaboration is planned. Initial analyses demonstrate that compared with BRAIN glioblastoma patients not enrolled in EX-TEM, those enrolled are younger (median age 58yrs vs 64yrs, *p* < 0.001), have a better performance status (ECOG 0–1 86 vs 80%, *p* < 0.001) and are more likely to have had subtotal resection than gross total resection (74 vs 64%, *p* < 0.001).

### Novel research activity – translational research combining tissue and registry data

BRAIN can capture the location of any biospecimen for each individual patient, facilitating retrieval for any translational research, along with the details of active research projects using this tissue. Further work is underway to broaden these data fields and to develop a patient-entered consent module, allowing patients to consent to release of tissue and clinical data for research purposes, and storing this consent centrally. Together, this will create a unique resource and drive efficiency, enabling researchers to locate tissue samples for various patient subgroups of interest.

**Fig. 2:**
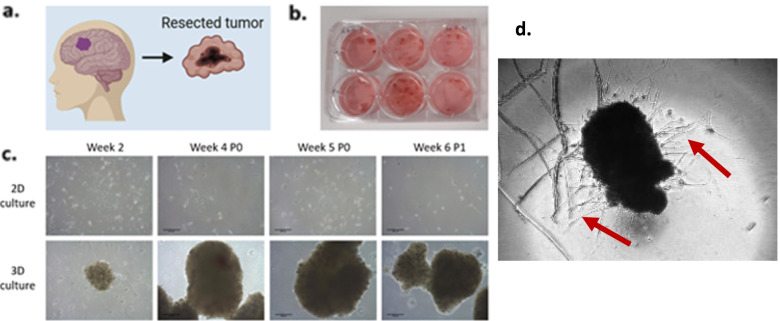
Representative brain cancer workflow for 3D organoid and 2D cell culture generation. **a**, **b** Resected tumour tissue is received and processed in the laboratory within 4 h. **c** 2D and 3D cultures are established, with first passage occurring at ~ 5 weeks. **d** Glioblastoma PDTO image demonstrating typical invasion

BioBRAIN is a translational program that formalises a focus on collecting fresh tumour tissue and blood samples, alongside the comprehensive clinical data captured in BRAIN, with tissue analysis and data to be combined to support a range of research efforts. The initial focus is developing glioblastoma patient-derived tumour organoids (PDTO) and patient-derived xenograft (PDX) models. These will enable an array of research efforts, including the discovery of novel therapies, biomarker discovery and validation, and assessment and understanding of therapy response and resistance. The PDTOs will also support prospective trials where treatment selection is tailored based on in vitro chemosensitivity testing, similar to our existing laboratory program in colorectal cancer (ACTRN12620001353987). BioBRAIN has seen initial success in establishing and expanding organoids from all 10 surgical specimens received to date, both newly diagnosed and recurrent glioblastoma using our ‘refined’ Jacob method [25] (see Fig. 2), including delayed culture from frozen and biobanked samples, with drug testing of these now underway (unpublished data).

## Discussion

Here we report on the first Australian data collection effort in brain tumours, which we believe to be unique worldwide given the number of sites and patients involved and the extent to which the registry resource is being leveraged to support clinical and translational research. The enthusiasm and support from contributing sites has been demonstrated by the uptake and patient recruitment in a short time span. Further sites continue to be added, including sites across Australia and overseas, with the first international site in the United Kingdom, and the broader interest to date confirms the value of BRAIN data collection and the leveraged research potential of this unique resource.

Ultimately, the greatest challenge for any RWD effort is maintaining a high standard of data quality. Incomplete or inaccurate data is particularly a risk when there is limited monitoring or maintenance of data, which will inevitably compromise the quality of data obtained and research output [26]. BRAIN has been designed to optimise data quality. Data completeness and data accuracy are augmented through (1) the use of inbuilt logic and completion metrics with warning messages and visual cues; (2) a comprehensive user guide and data dictionary to ensure consistency in data capture; (3) supervised training for all data entry personnel, such as medical staff, clinical trials staff or data officers; and (4) regular central data audits and cleaning for logical inconsistencies with feedback to sites. With the aim of close to real-time data collection where feasible (so missing data can be chased and captured efficiently) and access to source data at multiple locations (including patient letters or private specialist rooms), BRAIN has enabled high quality RWD collection to best support audit and research.

EX-TEM is the world’s first registry trial in neuro-oncology and one of the first in any cancer type in Australia. Registry trials are a novel research methodology that can overcome many of the challenges associated with conducting traditional stand-alone randomised controlled trials (RCT), such as uncertain external validity, substantial infrastructure and funding requirements, and siloed data collection efforts [27]. Registry trials typically have much broader inclusion criteria, allowing enrolment of real-world patients and use existing data registries to identify and recruit patients, collect trial-related data including adverse events and gather follow-up data including survival outcomes [26]. The pivotal TASTE trial in cardiology [28], published in 2013, demonstrated that the registry trial model was (1) feasible, recruiting over 7000 patients (60% of all eligible patients), (2) time efficient, completing within 3 years (a feat less than 10% of RCTs manage to achieve [29]), and (3) cost effective, estimating a 90% cost saving compared with a conventional RCT [26]. EX-TEM has also recruited patients across metropolitan and regional Australia, demonstrating the viability of this approach even at sites that manage only a small number of patients, and collects toxicity data, which is traditionally difficult to collect in registries, through a trial-specific adverse event module. Further registry trials in oncology are underway in Australia utilising disease-specific cancer registries (REAL-PRO: ANZCTRN12620000463976; ALT-TRACC: ANZCTRN12618001480279), with further concepts being explored for BRAIN.

BioBRAIN is a dedicated brain tumour translational program linked with BRAIN and provides a pipeline for biospecimen and linked data collection. It is now well established at two independent clinical sites with more to come on board. Compared with a ‘one-size-fits-all’ approach, biomarker directed therapy has revolutionised standards of care for many cancer types, such as HER2-directed therapy in breast cancer and small molecule inhibitors in non-small cell lung cancer [30–33]. To date the same degree of personalisation has not been achieved in patients with brain cancer, where tailoring treatment according to emerging biomarkers for glioblastoma has yet to lead to improvements in survival [34, 35]. Further exploration of pre-clinical models is prudent to better understand the underlying mechanism for resistance and therapy failure, potentially informing clinical management. BRAIN facilitates access to available tumour samples for each patient, linked with comprehensive clinical data through the use of common and unique patient identifiers. In the future, this will form the basis of a unique tissue registry accessible to the wider research community.

Ultimately BRAIN is designed to maximise the value that can be derived from RWD, with a focus on using and reusing the same data across many projects. Further data quality improvements are being explored including a hybrid model incorporating passive data flow through linkages with electronic medical records, as well as the role of artificial intelligence (AI) to prompt staff to complete BRAIN data entry at the time of major developments. In addition, high quality and comprehensive data can support almost limitless research initiatives. Examples include supporting the collection of patient-reported outcomes (PRO) and quality of life data through a dedicated patient and carer portal, allowing the intersection of PRO-clinical-translational data to be explored; data linkages to support research in radiomics and health care utilisation; and the role of AI in clinical trial matching. Finally, this model of the development of BRAIN could be used to develop similar resources across other less common cancers or subtypes of common cancers.

## Conclusion

To our knowledge, this is the first initiative for brain tumour clinical data collection across many sites in Australia, soon to be an international effort. We have described the design and implementation process, and the uptake of the registry, as well as the substantial research potential, including translational research studies and prospective registry-based trials. Ultimately, BRAIN has the capacity to collect comprehensive clinical and translational data on all patients with brain tumours in Australia and beyond. These data can be used to describe practice patterns and outcomes for patients in Australia, identify relevant predictive and prognostic markers, facilitate translational research and registry-based trials and drive research efficiency. However, the promise is also much more. Facilitating the collection of data on large numbers of patients leads to impactful research at centres across Australia, ensuring that brain tumour patients will receive the best care possible and pave the way for future discoveries.

## Supplementary Information

**Additional file 1: Supplementary Table 1.** Data points collected in BRAIN. **Supplementary Figure 1.** Schematic overview of BRAIN with surrounding tumour types representing predetermined modules.

## Data Availability

The datasets generated and/or analyzed to support the findings of this study are contained within the BRAIN registry, but are not publicly available due to confidentiality, security and ownership matters. They may be available from the corresponding author upon reasonable request.
