# Supplementary material for: Beyond standard data collection – the promise and potential of BRAIN (Brain tumour Registry Australia INnovation and translation registry)
Source: BMC Cancer. 2022 Jun 2;22:604. doi: 10.1186/s12885-022-09700-3 (PMC9161524; doi:10.1186/s12885-022-09700-3)
Supplement: Supplementary file 1 — Additional file 1: Supplementary Table 1. Data points collected in BRAIN. Supplementary Figure 1. Schematic overview of BRAIN with surrounding tumour types representing predetermined modules. [file 12885_2022_9700_MOESM1_ESM.docx]

**Supplementary Table 1: Data points collected in BRAIN**

| ***Detail*** | ***Data fields*** |
| --- | --- |
| *Patient details* | Date of birth  Gender  Indigenous status  Country of birth  Language  Medicare number  Postcode |
| *Treatment location* | Clinicians  Primary hospital  Type of institutions  Hospital site(s) of ongoing care |
| *History* | Date of diagnosis  ECOG performance status  Past history of CNS tumour or other cancer |
| *Preoperative details* | Preoperative investigations  Hemisphere and location of tumour  Number of tumours  Size of tumour  Treatment planned |
| *Surgery details* | Previous biopsy date  Date of surgery  Surgeon  ASA score  Operation performed  Extent of resection  Involvement in clinical trial  Dates of admission  Delays in discharge  Surgical/medical complications  Return to theatre  Site of ongoing care |
| *Histopathology details* | WHO grade  Histology  Biomarkers (genetic/epigenetic) relevant to each tumour type  Extent of extracranial disease if applicable |
| *Chemotherapy/Radiotherapy* | Dates of treatment  Location  Chemotherapeutic agent  Type, fractions and dose of radiotherapy  Toxicities/dose reduction  Reasons for stopping therapy  Enrolment in clinical trial |
| *Outcome details* | Date of follow up  Vital status  Disease status at last visit  Medications at last visit |
| *Translational details* | Tissue stored  Projects involved |

**
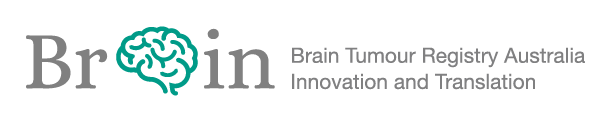
****Supplementary Figure 1: Schematic overview of BRAIN with surrounding tumour types representing predetermined modules**

**Follow-up**

Disease status

Medications

Vital status

**Treatment**

Surgery

Chemotherapy

Radiotherapy

Recurrence/ progression

Readmission

Readmission

**Patient details**

Demographics

Medical History

Preoperative details

**Schwannoma**

**Metastasis**

**Glioma**

**Glioneuronal tumour**

**Medulloblastoma**

**Meningioma**

**Ependymoma**

**Acoustic Neuroma**

**Translational data**

**Trial specific module**

**Primary Cerebral Lymphoma**

**Pineal tumour**

**Germ cell tumour**

**Other (including but not limited to spinal cord tumours, benign lesions etc)**
